# Access to commercial destinations within the neighbourhood and walking among Australian older adults

**DOI:** 10.1186/1479-5868-9-133

**Published:** 2012-11-20

**Authors:** Andrea Nathan, Gavin Pereira, Sarah Foster, Paula Hooper, Dick Saarloos, Billie Giles-Corti

**Affiliations:** 1 Centre for the Built Environment and Health, School of Population Health, The University of Western Australia, Crawley, Australia; 2 McCaughey Centre: VicHealth Centre for Promotion of Mental Health and Community Wellbeing, School of Population Health, The University of Melbourne, Melbourne, Australia

**Keywords:** Physical activity, Walking, Built environment, Neighbourhood, Destinations, Objective measurement, Older adults, Seniors

## Abstract

**Background:**

Physical activity, particularly walking, is greatly beneficial to health; yet a sizeable proportion of older adults are insufficiently active. The importance of built environment attributes for walking is known, but few studies of older adults have examined neighbourhood destinations and none have investigated access to specific, objectively-measured commercial destinations and walking.

**Methods:**

We undertook a secondary analysis of data from the Western Australian state government’s health surveillance survey for those aged 65–84 years and living in the Perth metropolitan region from 2003–2009 (n = 2,918). Individual-level road network service areas were generated at 400 m and 800 m distances, and the presence or absence of six commercial destination types within the neighbourhood service areas identified (food retail, general retail, medical care services, financial services, general services, and social infrastructure). Adjusted logistic regression models examined access to and mix of commercial destination types within neighbourhoods for associations with self-reported walking behaviour.

**Results:**

On average, the sample was aged 72.9 years (SD = 5.4), and was predominantly female (55.9%) and married (62.0%). Overall, 66.2% reported some weekly walking and 30.8% reported sufficient walking (≥150 min/week). Older adults with access to general services within 400 m (OR = 1.33, 95% CI = 1.07-1.66) and 800 m (OR = 1.20, 95% CI = 1.02-1.42), and social infrastructure within 800 m (OR = 1.19, 95% CI = 1.01-1.40) were more likely to engage in some weekly walking. Access to medical care services within 400 m (OR = 0.77, 95% CI = 0.63-0.93) and 800 m (OR = 0.83, 95% CI = 0.70-0.99) reduced the odds of sufficient walking. Access to food retail, general retail, financial services, and the mix of commercial destination types within the neighbourhood were all unrelated to walking.

**Conclusions:**

The types of neighbourhood commercial destinations that encourage older adults to walk appear to differ slightly from those reported for adult samples. Destinations that facilitate more social interaction, for example eating at a restaurant or church involvement, or provide opportunities for some incidental social contact, for example visiting the pharmacy or hairdresser, were the strongest predictors for walking among seniors in this study. This underscores the importance of planning neighbourhoods with proximate access to social infrastructure, and highlights the need to create residential environments that support activity across the life course.

## Background

The health benefits of a physically active lifestyle are comprehensive and well documented. Physical activity reduces the risk of cardiovascular disease, hypertension, stroke, type 2 diabetes, osteoporosis, obesity, some cancers, anxiety, and depression [1]. In addition, physical activity participation reduces risk of falls and fall-related injuries, and prevents or delays functional and mobility limitations in older adults (defined as ≥65 years of age in Australia) [2]. Yet, older adults are among the least physically active. Participation in sufficient amounts of physical activity to accrue health benefits, defined as at least 30 minutes of moderate-intensity activity on most days of the week [3], remains low around the world. In Western Australia, 52.5% of adults aged over 45 years are sufficiently active, and this further reduces to only 30.1% among those aged ≥80 years [4]. Using objective physical activity data obtained with accelerometers, Troiano and colleagues reported just 2.4% of the U.S. population aged 60+ to be sufficiently active [5]. With the population ageing, the importance of promoting and encouraging older adults to be physically active will only grow in public health significance.

For older adults, one of the most popular forms of physical activity undertaken is walking [6]. It is highly accessible, low in cost, can be easily integrated into daily routines, and often occurs on neighbourhood streets and in public areas within the neighbourhood [7]. The importance of the neighbourhood environment in which people live and how it impacts walking is consistent with social-ecological models of behaviour. Such frameworks posit that multiple levels of interacting factors within a person’s surrounds will influence their behaviour [8,9]. In other words, individual factors (e.g., demographic characteristics), interpersonal relationships (e.g., social networks and social support systems), physical environment factors (e.g., built and natural environments), and public policy factors (e.g., laws and regulations) work synergistically to influence walking. Thus, to increase population physical activity patterns, attention must be given towards the implementation of multi-level interventions [10].

Researchers have shown neighbourhood walkability, a composite measure of residential density, street connectivity, and land-use mix characteristics, to be related to walking among older adults [11-13]. But for research findings to be translated into policy and practice, more detailed information is required, particularly in terms of specific types and mixes of neighbourhood destinations that may be necessary to support walking. Only a handful of studies have examined access to specific neighbourhood destinations (e.g., public transport, post boxes, and convenience or grocery stores) [14-17]. Yet, the types of neighbourhood destinations associated with walking may differ across the life course. For example, access to schools may be important for children and workplaces salient for employed adults, but have little relevance for retired, older adults. Research involving older adults has considered the total number of neighbourhood destinations [18-20], but only one study among older women, which utilised self-reported environmental data, has examined access to specific destinations within the neighbourhood and walking [21]. The relationship between walking and the presence of a convenience, deli, or grocery store within a 20 minute walk from home approached statistical significance, whereas living within walking distance of a biking or walking trail, park, and department, discount or hardware store were significantly related to daily pedometer steps among older women [21]. It is possible that access to neighbourhood destinations differentially relates to walking according to gender. In addition, the aforementioned study used self-reported perceptions to assess the presence of destinations [21]. Others report poor concordance between self-report and objective environmental measures, though environmental attributes measured using both types were independently associated with walking, suggesting that the measures capture different dimensions of neighbourhood environments [22,23]. With scant evidence considering objective environmental measures, research is needed to investigate whether the presence of specific, objectively-measured local destinations relates to older adults’ walking. The aim of this study was to examine associations between access to and mix of commercial destinations within the neighbourhood and walking in a sample of older adults living in Perth, Western Australia.

## Methods

This research involved a secondary cross-sectional analysis of data from the Health and Wellbeing Surveillance System co-ordinated by the state government’s Department of Health. Briefly, the surveillance system surveys people living in Western Australia by Computer Assisted Telephone Interviews (CATI) to collect self-reported data on health behaviours and levels and patterns of associated risk and protective factors across the life course [24]. Conducted monthly since 2002, 550 households are randomly selected from a stratified sampling frame each month, with annual response rates ranging from approximately 80-84%. Sample data are weighted to account for over-sampling and the probability of selection, thus ensuring representativeness of the state’s population [24]. For the present study, we utilised data collected between 2003 and 2009 for residents of the Perth metropolitan region who were aged 65–84 years at the time data were gathered (n = 2,918). Ethical approval was provided by the Department of Health Western Australia Human Research Ethics Committee.

### Self-reported walking

Within the surveillance system, physical activity behaviour is assessed using the widely accepted Active Australia Survey [25]. This tool has acceptable convergent validity for community-dwelling older adults [26]. Based on public health recommendations for physical activity [2,3], items on frequency and total duration of walking for recreation, exercise or to get to or from places were used to compute two dichotomous dependent variables: prevalence of weekly walking (none vs. some); and sufficient minutes of walking per week (insufficient [i.e., <150 minutes] vs. sufficient [i.e., ≥150 minutes]).

### Objective neighbourhood destinations

The full household address for each participant had previously been geocoded by the Department of Health as part of the surveillance system [24], enabling objective environmental data to be linked using a Geographic Information System (GIS). Individual-level neighbourhood service areas were produced based on 400 m and 800 m road network distances from participants’ home address, distances informed by the literature [18,19,27,28].

Comprehensive data on neighbourhood destinations were purchased from Sensis Pty. Ltd. – the data custodians of the Australian Yellow Pages — for three time points (2004, 2005, and 2007). This allowed us to match the most temporally relevant spatial data to the surveillance data. In sum, Sensis data from 2004 were matched to participants surveyed from February 2003 to June 2005, 2005 data matched to those surveyed from July 2005 to December 2006, and 2007 data for the remaining participants. Individual destinations were grouped into six mutually exclusive categories according to domain: food retail (e.g., delicatessen, supermarket); general retail (e.g., newsagent, shopping centre); medical care services (e.g., doctor, medical centre); financial services (e.g., bank, post office); general services (e.g., hairdresser, pharmacy); and social infrastructure (e.g., café or restaurant, church or place of worship).

Access to each commercial destination type was specified for both the 400 m and 800 m neighbourhood service areas. In addition, the number of commercial destination types present within each service area was summed to examine the mix or diversity of accessible commercial destinations within each participant’s neighbourhood.

### Covariates

Participants reported age, sex, highest education level attained, marital status, self-rated health [29], and use of assistive equipment (i.e., having a health problem requiring use of a cane, wheelchair, special bed or telephone etc.).

To account for potential confounding effects, street connectivity was calculated as the count of three (or more) intersections divided by the area (m^2^) of each participant’s service area. This was computed for both the 400 m and 800 m neighbourhood service areas, with values standardised as z scores across the sample.

### Statistical analysis

Analyses were conducted using SAS v9.2. Logistic regression models examined the prevalence of some walking and engagement in sufficient minutes of walking, and relationships with access to and mix of commercial destination types within 400 m and 800 m neighbourhood service areas. In addition, the moderating effect of sex was investigated by including the cross-product term within the model, and then conducting stratified analyses to interpret any significant interactions. All models progressively adjusted for demographic covariates (i.e., age, sex, highest education level, marital status, self-rated health, and use of assistive equipment) and street connectivity within the service area. P values less than 0.05 were considered statistically significant.

## Results

Demographic characteristics for the study sample (n = 2,918) are presented in Table 1. In summary, most participants were aged 65–74 years (61.8%), were female (55.9%), and were married (62.0%). Approximately 12% of the sample rated their health as ‘excellent’, while a similar proportion used assistive equipment to aid health conditions. Overall, 66.2% of participants reported engaging in some weekly walking. However, most of them (69.2%) reported <150 minutes per week, with only 30.8% engaging in sufficient walking.

**Table 1:** Descriptive statistics for sample (n = 2,918)

**Demographic characteristic**	**n**	**%**
Age: 65–74 years	1802	61.8
75-84 years	1116	38.2
Sex: Male	1287	44.1
Female	1631	55.9
Highest education level ^a^: Secondary or less	1437	49.2
TAFE/Trade qualification	1055	36.2
Tertiary degree or equivalent	387	13.3
Marital status ^b^: Married/De facto relationship	1810	62.0
Separated/Divorced/Never married	374	12.8
Widowed	732	25.1
Self-rated health: Poor	153	5.2
Fair	452	15.5
Good	1034	35.4
Very good	935	32.0
Excellent	344	11.8
Use of assistive equipment ^c^: No	2571	88.1
Yes	347	11.9
**Self**-**reported walking**	**n**	**%**
Some weekly walking: No	986	33.8
Yes	1932	66.2
Sufficient minutes of weekly walking ^d^: No	2019	69.2
Yes	899	30.8

TAFE = technical and further education, ^a^ n = 39 missing data. ^b^ n = 2 missing data. ^c^ defined as having a health problem requiring use of a cane, wheelchair, special bed or telephone etc. ^d^ defined as ≥150 weekly minutes of walking.

As seen in Table 2, the most common type of commercial destination accessible within 400 m of participants’ home was medical care services (27.2%). Approximately one half of the sample lived within 800 m of food retail (50.0%) and general services (51.3%), while 58.0% had access to social infrastructure within the 800 m neighbourhood service area. In terms of the mix of destination types accessible within 400 m and 800 m neighbourhood service areas, mean scores were 1.0 (SD = 1.5, range = 0-6) and 2.8 (SD = 2.1, range = 0-6) respectively.

**Table 2:** Proportion of sample with access to commercial destinations within 400 m and 800 m neighbourhood service areas

**Destination access**	**400 m service area**		**800 m service area**	
	**n**	**%**	**n**	**%**
Food retail ^a^	500	17.1	1459	50.0
General retail ^b^	336	11.5	1136	38.9
Medical care services ^c^	794	27.2	1806	61.9
Financial services ^d^	113	3.9	515	17.6
General services ^e^	525	18.0	1498	51.3
Social infrastructure ^f^	630	21.6	1692	58.0

^a^ includes bakery, butcher, delicatessen, green grocer, liquor outlet, petrol station store, supermarket. ^b^ includes art/craft store, bookstore, general store, hardware store, newsagent, shopping centre, toy/hobby store. ^c^ includes doctor, medical centre. ^d^ includes bank, post office. ^e^ includes beauty salon, hairdresser, pharmacy. ^f^ includes art gallery, café or restaurant, church or place of worship, cinema, community hall, fast food outlet, hotel or tavern.

Table 3 presents the unadjusted and adjusted odds ratios examining access to and mix of commercial destinations within 400 m and 800 m neighbourhood service areas associated with some walking. After adjustment for demographic characteristics, access to general services (i.e., hairdresser or pharmacy) within 400 m (OR = 1.33, 95% CI = 1.07–1.66, p = 0.011) and 800 m (OR = 1.20, 95% CI = 1.02–1.42, p = 0.027) were both positively related to participation in some walking. Also, older adults were 1.19 times more likely to engage in some walking when social infrastructure, such as a café or restaurant, or church or place of worship, were present within the 800 m neighbourhood service area (95% CI = 1.01-1.05, p = 0.043). Street connectivity was significantly associated with some walking within both 400 m (OR = 1.12, 95% CI = 1.02-1.22, p = 0.015) and 800 m (OR = 1.11, 95% CI = 1.02-1.22, p = 0.019) service areas. With further adjustment for street connectivity, only access to general services within 400 m remained significantly associated with some walking (OR = 1.29, 95% CI = 1.03-1.61, p = 0.027), though associations for access to general services and social infrastructure within 800 m were still in a positive direction.

**Table 3:** Odds ratios examining access to and mix of commercial destinations associated with some walking

**Destination access within 400 m service area**	**Unadjusted**			**Adjusted for demographics**^a^			**Adjusted for connectivity**^b^		
	**OR**	**95%CI**	**p**	**OR**	**95%CI**	**p**	**OR**	**95%CI**	**p**
Food retail	1.02	0.83, 1.25	0.839	1.05	0.84, 1.30	0.676	1.01	0.81, 1.26	0.933
General retail	1.02	0.80, 1.30	0.851	1.00	0.77, 1.29	0.979	0.95	0.73, 1.24	0.715
Medical care services	0.94	0.79, 1.11	0.444	0.96	0.80, 1.15	0.653	0.91	0.76, 1.10	0.337
Financial services	1.05	0.70, 1.57	0.810	1.10	0.71, 1.70	0.675	1.03	0.66, 1.60	0.900
General services	**1**.**33**	**1**.**08**, **1**.**63**	**0**.**007**	**1**.**33**	**1**.**07**, **1**.**66**	**0**.**011**	**1**.**29**	**1**.**03**, **1**.**61**	**0**.**027**
Social infrastructure	1.05	0.87, 1.27	0.576	1.02	0.83, 1.24	0.884	0.98	0.80, 1.20	0.835
**Destination mix within 400 m service area**^c^	1.02	0.97, 1.07	0.461	1.02	0.97, 1.08	0.473	1.01	0.95, 1.06	0.851
**Destination access within 800 m service area**									
Food retail	1.04	0.90, 1.22	0.584	0.98	0.83, 1.15	0.767	0.94	0.80, 1.12	0.499
General retail	1.03	0.88, 1.20	0.757	1.00	0.85, 1.18	0.996	0.97	0.82, 1.15	0.738
Medical care services	1.05	0.90, 1.23	0.559	1.01	0.85, 1.20	0.893	0.96	0.81, 1.14	0.645
Financial services	0.96	0.78, 1.17	0.683	0.93	0.75, 1.15	0.507	0.90	0.73, 1.12	0.342
General services	**1**.**25**	**1**.**07**, **1**.**46**	**0**.**005**	**1**.**20**	**1**.**02**, **1**.**42**	**0**.**027**	1.16	0.98, 1.38	0.079
Social infrastructure	**1**.**19**	**1**.**02**, **1**.**39**	**0**.**028**	**1**.**19**	**1**.**01**, **1**.**40**	**0**.**043**	1.15	0.97, 1.36	0.108
**Destination mix within 800 m service area**^c^	1.03	0.99, 1.07	0.147	1.02	0.98, 1.06	0.395	1.01	0.97, 1.05	0.779

OR = odds ratio; CI = confidence interval. Statistically significant results (p < 0.05) marked in bold typeface. ^a^ Model adjusted for age, sex, highest education level, marital status, self-rated health, and use of assistive equipment. ^b^ Model adjusted for demographic characteristics and street connectivity within service area. ^c^ Summed score for number of accessible commercial destination categories, range 0–6.

Unadjusted and adjusted odds ratios examining access to and mix of commercial destinations within 400 m and 800 m neighbourhood service areas and associations with sufficient walking are presented in Table 4. No significant differences were found for street connectivity within 400 m or 800 m services areas and sufficient walking. The only commercial destination type significantly associated with sufficient walking was medical care services, which reduced the odds of sufficient walking when accessible within 400 m (OR = 0.77, 95% CI = 0.63-0.93, p = 0.008) and 800 m (OR = 0.83, 95% CI = 0.70-0.99, p = 0.044) respectively and adjusted for both demographic characteristics and street connectivity. Sex did not significantly moderate any of the associations examined (results not shown).

**Table 4:** Odds ratios examining access to and mix of commercial destinations associated with sufficient walking

**Destination access within 400 m service area**	**Unadjusted**			**Adjusted for demographics**^a^			**Adjusted for connectivity**^b^		
	**OR**	**95%CI**	**p**	**OR**	**95%CI**	**p**	**OR**	**95%CI**	**p**
Food retail	0.87	0.70 ,1.08	0.200	0.86	0.69, 1.08	0.188	0.88	0.70, 1.10	0.246
General retail	0.86	0.67, 1.10	0.232	0.80	0.62, 1.05	0.107	0.82	0.63, 1.07	0.144
Medical care services	**0**.**78**	**0**.**65**, **0**.**93**	**0**.**006**	**0**.**76**	**0**.**63**, **0**.**92**	**0**.**005**	**0**.**77**	**0**.**63**, **0**.**93**	**0**.**008**
Financial services	0.84	0.55, 1.29	0.429	0.85	0.55, 1.33	0.480	0.89	0.57, 1.39	0.602
General services	1.04	0.85, 1.27	0.734	1.00	0.81, 1.24	0.971	1.02	0.82, 1.27	0.830
Social infrastructure	1.05	0.87, 1.27	0.633	1.02	0.83, 1.24	0.861	1.04	0.85, 1.27	0.721
**Destination mix within 400 m service area**^c^	0.97	0.92, 1.02	0.201	0.96	0.90, 1.01	0.124	0.96	0.91, 1.02	0.187
**Destination access within 800 m service area**									
Food retail	0.92	0.79, 1.08	0.316	0.87	0.74, 1.03	0.109	0.88	0.74, 1.04	0.126
General retail	0.93	0.79, 1.09	0.366	0.90	0.76, 1.07	0.248	0.91	0.77, 1.08	0.284
Medical care services	0.87	0.74, 1.02	0.092	**0**.**83**	**0**.**70**, **0**.**99**	**0**.**036**	**0**.**83**	**0**.**70**, **0**.**99**	**0**.**044**
Financial services	0.96	0.78, 1.18	0.700	0.96	0.77, 1.20	0.738	0.97	0.78, 1.21	0.811
General services	1.09	0.93, 1.28	0.279	1.06	0.89, 1.25	0.526	1.07	0.90, 1.27	0.441
Social infrastructure	0.91	0.78, 1.07	0.246	0.90	0.76, 1.06	0.194	0.90	0.76, 1.07	0.227
**Destination mix within 800 m service area**^c^	0.98	0.95, 1.02	0.357	0.97	0.94, 1.01	0.178	0.97	0.94, 1.02	0.216

OR = odds ratio; CI = confidence interval. Statistically significant results (p < 0.05) marked in bold typeface. ^a^ Model adjusted for age, sex, highest education level, marital status, self-rated health, and use of assistive equipment. ^b^ Model adjusted for demographic characteristics and street connectivity within service area. ^c^ Summed score for number of accessible commercial destination categories, range 0–6.

## Discussion

We examined associations between the presence of objectively-measured access to specific commercial destination types within the neighbourhood and older Australian’s walking, and found some differences in the types of commercial destinations associated with seniors’ walking compared with those generally reported among adults. This lends support to the need for policy-makers and practitioners to plan or retrofit neighbourhood environments that support physical activity across the life course.

Our findings suggest that access to destinations providing more opportunities for social interaction – such as restaurants and religious institutions – and destinations enabling some incidental social contact on a more regular basis for older people – such as pharmacies and hairdressers – appear to be positively associated with walking among older adults. This follows some findings in adult populations, where closer proximity to restaurants and religious or cultural areas positively related to walking for transport-related purposes [16,17]. However, providing neighbourhood destinations where people can meet and engage with others may have important implications for the ageing population beyond physical health and walking. For example, access to proximate socially-based facilities has the potential to increase levels of social engagement and participation in retired older adults, who no longer have work-related social contact opportunities and who generally travel shorter distances from home [30,31]. Social activity is a key component of successful ageing [32], and is consistently linked with health and well-being [33-35]. This underscores the importance of planning neighbourhoods with proximate access to social infrastructure, not only for physical activity and health, but also for optimising the ageing process.

Retail destinations were found to be non-significantly related to walking in our sample. This is somewhat surprising and contrasts previous findings among adults, in which proximity to retail destinations such as local convenience stores, supermarkets or grocery stores, and newsagents are consistently and positively associated with transportation walking [14-17]. It is possible that proximity to retail destinations is less important for seniors compared with adults; however there are several other factors that warrant consideration. The purpose of travelling to retail destinations is to shop and purchase goods, and older adults may have less muscle strength to enable them in carrying their shopping home [1]. Furthermore, the additional weight associated with carrying shopping may reduce self-efficacy, which is an important mediator between fear of falling and functional ability [36]. Issues of self-efficacy may also relate to the quality of walking infrastructure, such as footpaths and the presence of benches or resting places along the route to retail destinations. The supportiveness or quality of neighbourhood environments for older adults’ walking has been previously reported [37].

We found the presence of medical care services to be negatively associated with sufficient walking, replicating the finding of Wang and Lee [19]. There may be some possible explanations for this, for example the reduced likelihood of walking may reflect unaccounted for self-selection bias. Seniors may purposefully seek to live in neighbourhoods with proximate access to medical care services, and planners and medical practitioners may intentionally locate medical care services in neighbourhoods with higher proportions of older adults. Though older adults are more likely to use such medical care services and to report them as being important destinations to access within the neighbourhood [38], others report that older adults have an increased propensity to carpool or be driven by family members when traveling to medical appointments [39]. It may be that even when medical care services are accessible within the neighbourhood, it is not the type of destination in which someone would necessarily walk when physically unwell and in need of medical care. Moreover, medical care services may occupy such large land parcels that other destinations, which older adults may indeed walk to, cannot also be located in the area. It is also important to note that the older adult population is not homogenous, and the needs of the ‘young-old’ and ‘old-old’ may differ greatly in terms of the importance of close proximity to medical care services and whether or not they walk to this destination type. Future research should consider self-selection effects and age moderation effects when examining commercial destinations of importance across the life course.

When adjusting for the effects of street connectivity, we found access to destinations within the 400 m neighbourhood service areas to remain significantly associated with walking, however within the 800 m service areas, previously positive relationships between walking and access to general services and social infrastructure were no longer significant. It is worth noting that street connectivity and access to destinations are related, as a more connected street network encompasses more possible routes along the street network, increasing the area or size of service areas, and larger services areas are more likely to have destinations present within them. Nonetheless, the findings suggest that street connectivity may not impact the association between destinations and shorter walks (i.e., within 400 m), as much as it does for longer walks (i.e., within 800 m). It may be that street connectivity plays a different sort of role in influencing walking when destinations are located further away, and may mediate relationships between walking and access to destinations within 800 m. Evidence supporting the role of street connectivity on physical activity in older adults is mostly mixed, with some reporting positive associations [40-42], others reporting negative associations [20,43,44], and still others reporting no relationship [18,40,45]. This may be because the importance of street connectivity, and the direction of its influence, may be dependent upon the environmental scale considered. However, it is also possible that other attributes may be important to consider when examining access to destinations within 800 m service areas. For example, perceptions of distance and the directness of possible walking routes may contribute to the attenuating associations at 800 m. Future research considering objective and perceived measures of the same built environment attributes at the same environmental scale, and then across varying environmental scales, would assist in attempts to further disentangle relationships.

### Study limitations

The current study has several limitations to consider. While we attempted to categorise commercial destinations by type or domain, it is possible that these require further specification and understanding, based on the purpose and frequency for which older adults visit such destinations. For example, fast-food outlets have been categorised as food retail in previous studies [46,47], but we chose to include it within the social infrastructure category based on findings among older adults [48]. It is also possible that food retail requires further specification in that supermarkets or grocery stores are probably visited more often than other types of food retail destinations. In addition, destinations that are frequently used by older adults (e.g., bank, post office, supermarket) generally had low proportions in this study, indicating that our sample had poor access to commercial destination types overall.

Other limitations include the cross-sectional design, which limits causality, and the influence of self-selection bias cannot be discounted. Also, utilising existing data from a surveillance system was a limitation in that our behavioural outcome measures were self-reported and assessed total, not purpose specific, walking. The influence of neighbourhood environmental attributes on walking appears to differ according to walking purpose, i.e., for recreational walking and transport walking [49], and the need for measures that are context and behaviour specific has been previously highlighted [50]. This may explain why many commercial destination types within the neighbourhood were not associated with walking in our study.

## Conclusions

Commercial destinations within the neighbourhood that promote walking in an older population appear to differ from those among adult samples. Destinations allowing opportunities for more social interaction, be that more purposeful (e.g., visiting a café or restaurant, church involvement), or incidental (e.g., visiting the hairdresser), appeared to encourage seniors’ walking. Neighbourhood environments with access to proximate social destinations may not only promote walking and physical activity, but also help ensure older residents remain socially engaged with the local community. In sum, our findings highlight the need to plan residential environments that are supportive of all age groups in society.

## Competing interests

The authors declare that they have no competing interests.

## Authors’ contributions

AN contributed to study conception and design. GP conducted the analysis. AN, GP, SF interpreted data and drafted the manuscript. PH contributed to study conception and critically revised drafts. DS, BGC designed the overall study and critically revised drafts. All authors read and approved the final manuscript.
